# Comparative Study of Structural Changes Caused by Different Substitutions at the Same Residue on α-Galactosidase A

**DOI:** 10.1371/journal.pone.0084267

**Published:** 2013-12-26

**Authors:** Seiji Saito, Kazuki Ohno, Hitoshi Sakuraba

**Affiliations:** 1 Department of Medical Management and Informatics, Hokkaido Information University, Ebetsu, Hokkaido, Japan; 2 Department of Research, Not-for-Profit Organization for the Promotion of Research on Intellectual Property Tokyo, Chiyoda, Tokyo, Japan; 3 Department of Clinical Genetics, Meiji Pharmaceutical University, Kiyose, Tokyo, Japan; Russian Academy of Sciences, Institute for Biological Instrumentation, Russian Federation

## Abstract

Missense mutations in the α-galactosidase A (GLA) gene comprising the majority of mutations responsible for Fabry disease result in heterogeneous phenotypes ranging from the early onset severe “classic” form to the “later-onset” milder form. To elucidate the molecular basis of Fabry disease from the viewpoint of structural biology, we comprehensively examined the effects of different substitutions at the same residue in the amino acid sequence of GLA on the structural change in the enzyme molecule and the clinical phenotype by calculating the number of atoms affected and the root-mean-square-distance value, and by coloring of the atoms influenced by the amino acid replacements. The results revealed that the severity of the structural change influences the disease progression, i.e., a small structural change tends to lead to the later-onset form and a large one to the classic form. Furthermore, the study revealed the residues important for expression of the GLA activity, i.e., residues involved in construction of the active site, a disulfide bond or a dimer. Structural study from such a viewpoint is useful for elucidating the basis of Fabry disease.

## Introduction

Fabry disease (MIM 301500) is an X-linked genetic disorder resulting from a deficiency of α-galactosidase A (GLA; EC 3.2.1.22) activity [1]. GLA deficiency causes the progressive accumulation of glycolipids, predominantly globotriaosylceramide, in lysosomes of cells. The disease exhibits a wide range of clinical phenotypes, from the early-onset severe “classic” form to the “later-onset” milder one [2]. Generally, male patients with the classic form of Fabry disease, who have little or no GLA activity, develop pain in the peripheral extremities, hypohidrosis, angiokeratomas and corneal opacities in childhood or adolescence, and manifest renal, cardiac, and cerebrovascular complications in the fourth to fifth decade of life [3]. On the other hand, male patients with the later-onset form, who have residual GLA activity, develop heart and kidney disorders without the childhood symptoms [4]. Heterozygous Fabry females exhibit a wide spectrum of disease severity ranging from asymptomatic to presentation with the classic disease due to random X-chromosomal inactivation [5].

The *GLA* gene is localized to Xq22.1 and encodes a precursor GLA comprising a 429-amino acid polypeptide, the enzyme being glycosylated and then processed to the mature form comprising 398 amino acids, and it exists as a homodimer in lysosomes [1]. Each monomer contains a (β/α)_8_ barrel domain containing the active site and an anti- parallel β-sheet domain [6]. So far, more than 600 genetic mutations causing Fabry disease have been identified, and it is known that gross alterations, nonsense mutations, and most of the splicing mutations of the *GLA* gene lead to the classic form. However missense mutations comprising the majority of mutations result in heterogeneous phenotypes ranging from the classic form to the later-onset one.

Previously, Garman and his research group determined the GLA structure by means of X-ray crystallography and analyzed the locations of missense and nonsense mutations in the three-dimensional structure [6], [7]. Our research group studied structural changes caused by missense mutations responsible for Fabry disease by calculating the numbers of affected atoms and the root-mean-square distance (RMSD) values [8], and proposed a phenotype prediction model based on sequential and structural information [9].

In this study, we comprehensively examined different substitutions at the same residue in the amino acid sequence of GLA, focusing on their effects on the structural change in the enzyme protein and the clinical phenotype, as such investigation will provide us with information about the relationship between the enzyme structure and the disease.

## Materials and Methods

### GLA missense mutations

We collected GLA missense mutations and polymorphisms registered on the Human Gene Mutation Database (http://www.hgmd.cf.ac.uk/) and Fabry database (http://fabry-database.org/). From them, we selected cases in which more than two substitutions at the same residue in the amino acid sequence of GLA have been reported. Finally, we analyzed 157 amino acid substitutions at 67 residues in this study.

### Structural modeling of mutant GLAs

Structural models of mutant GLA monomers were built by means of homology modeling using molecular modeling software, TINKER (http://dasher.wustl.edu/tinker/) [10]–[14]. The crystal structure of human GLA (PDB: 1R46) [6] was used as a template, and energy minimization was performed. The root-mean-square gradient value was set at 0.05 kcal/mol·Å.

### Calculation of the number of atoms influenced by an amino acid substitution and the RMSD values between the wild type GLA and mutant GLAs

Each mutant model was superimposed on the wild type GLA structure based on the Cα atoms by the least-square-mean fitting algorithm, in which the optimal rotations and translations are found by minimizing the sum of the squared distances among all structures in the superposition [15]–[19]. We defined that the atom was affected by an amino acid substitution when the position of the atom in a mutant differed from that in the wild type structure by more than 0.15 Å. We calculated the numbers of atoms affected in the main chain and in the side chain of the enzyme, and in the active site (E170 and E231). Then, we calculated the RMSD values between the wild type GLA and mutant GLAs [15]–[19].

### Determination of the solvent-accessible surface area (ASA) value

The ASA value of an amino acid residue in the wild type GLA was calculated using Stride (http://webclu.bio.wzw.tum.de/stride/) to evaluate the location of the residue in the GLA molecule.

### Coloring of the atoms influenced by an amino acid substitution

To determine the influence of the amino acid substitutions geographically and semi- quantitatively, coloring of the influenced atoms in the three-dimensional structure of the enzyme molecule was performed for 12 mutants (M72I, M72R, M72V, E66G, E66K, E66Q, C56G, C56F, C56Y, W236C, W236L, and W236R) as to four positions (M72, E66, C56, and W236) in the GLA structure. The colors of affected atoms were shown on the basis of the distance between the wild type and mutant one.

### Statistical analysis

To determine the differences in the number of the affected atoms and the RMSD value between the classic Fabry group and later-onset one, statistical analysis was performed using Excel 2013 (Microsoft, Redmond, WA) by means of one side Welch's *t* test, it being taken that there was a significant difference if p<0.05. Then, power analysis (http://www.statmethods.net/stats/power.html) was performed using G*POWER3 to evaluate statistical power for this Welch's *t* test [20]. In power analysis calculation, sample sizes of two groups and significant level were set to 134, 11, and 0.05, respectively.

## Results

### Different substitutions at the same residue in the amino acid sequence of GLA

We examined the numbers of affected atoms for the whole enzyme protein and for the active site, and the RMSD and ASA values. The results are shown in Table 1. The numbers of atoms affected in the main chain and in the side chain, and the RMSD values in the classic Fabry group were 107±129 (134), 131±152 (134), and 0.089±0.074 Å (134), respectively. The values are expressed as average ± standard deviation (number of cases). On the other hand, in the later-onset Fabry group, they were 23±36 (11), 28±50 (11), and 0.033±0.038 Å (11), respectively. The statistical analysis showed significant differences between the classic Fabry group and the later-onset Fabry group in numbers of affected atoms in the main chain (P<0.001, Welch's *t* test) and in the side chain (P<0.001, Welch's *t* test), and RMSD (P<0.001, Welch's *t* test). The results of the power analysis revealed that the estimated values of power were 0.70, 0.72, and 0.80 for numbers of affected atoms in the main chain and in the side chain, and RMSD, respectively. This suggests that the structural change resulting from the amino acid substitutions leading to the classic phenotype is essentially greater than that in the later-onset one, although there are some exceptional cases, i.e., in R112H and R301Q, the numbers of affected atoms and the RMSD values are apparently large, although the patients with these mutations exhibited the later-onset phenotype (Table 1). Furthermore, the results revealed that there were no later-onset Fabry cases in which the structure of the active site was affected, although there were 57 affected cases among the 134 classic Fabry ones. This suggests that a defect of the active site tends to lead to the classic phenotype.

**Table 1:** Different substitutions at the same residue of the amino acid sequence of α-galactosidase A.

Genotype	Phenotype	Number of affected atoms			RMSD	ASA	Reference
		Main chain	Side chain	Active site	(Å)	(Å^2^)	
N34K	Classic	165	153	0	0.196	25.7	Hum Genomics 2006, 2: 297–309
N34S	Classic	6	1	0	0.029	25.7	Am J Hum Genet 1993, 53: 1186–97
P40L	Classic	80	83	0	0.111	0.8	J Invest Med 2000, 48: 227–35
P40S	Classic	15	12	0	0.032	0.8	FEBS Lett 1990, 259: 353–6
M42T	Classic	9	9	0	0.023	3.8	Mol Genet Metab 2002, 76: 23–30
M42V	Classic	12	13	0	0.028	3.8	Eur J Hum Genet 1996, 4: 219–24
G43R	Classic	413	471	12	0.177	0.0	Mol Med 2002, 8: 306–12
G43V	Classic	219	250	1	0.126	0.0	Mol Genet Metab 2002, 76: 23–30
H46R	Classic	25	27	0	0.043	0.0	Mol Med 1997, 3: 174–82
H46Y	Hetero	83	119	0	0.088	0.0	Hum Mutat 2001, 18: 459
R49C	Classic	125	136	0	0.109	55.3	Pharmacogenet Genomics 2008, 18: 773–80
R49G	Classic	182	197	0	0.169	55.3	Mol Med 2002, 8: 306–12
R49L	Classic	240	259	4	0.201	55.3	Hum Mol Genet 1994, 3: 667–9
R49P	Classic	174	209	2	0.125	55.3	Hum Mutat 2001, 18: 459
R49S	Classic	361	365	2	0.251	55.3	Eur J Hum Genet 1996, 4: 219–24
C52R	Classic	195	242	15	0.136	49.0	Hum Mutat 1996, 8: 38–43
C52S	Classic	2	1	0	0.022	49.0	Hum Mol Genet 1994, 3: 1795–9
C52Y	Classic	56	75	0	0.073	49.0	Biochim Biophys Acta 2010, 1802: 247–52
C56F	Classic	67	78	0	0.128	38.4	Hum Mol Genet 1994, 3: 1795–9
C56G	Classic	51	60	0	0.131	38.4	Am J Hum Genet 1993, 53: 1186–97
C56Y	Classic	58	65	0	0.132	38.4	Eur J Hum Genet 1996, 4: 219–24
E66G	Classic	45	74	0	0.062	29.2	Am J Hum Genet 2006, 79: 31–40
E66K	Classic	422	503	7	0.361	29.2	Hum Mutat 2005, 25: 412
E66Q	Polymorphism	23	32	0	0.048	29.2	Hum Genet 1992, 89: 29–32.
M72I	Classic	38	46	0	0.054	0.0	Mol Med 2002, 8: 306–12
M72R	Classic	145	198	1	0.119	0.0	Ned Tijdschr Geneeskd 200, 144: 2412–5
M72V	Later-onset	7	6	0	0.026	0.0	Hum Mutat 1998, Suppl 1: S213–16
L89P	Classic	6	12	0	0.023	0.0	Mol Med 1997, 3: 174–82
L89R	Classic	286	337	4	0.164	0.0	Hum Mol Genet 1994, 3: 1795–9
D92H	Classic	324	482	14	0.140	0.2	Eur J Hum Genet 1996, 4: 219–24
D92Y	Classic	408	580	16	0.182	0.2	Mol Med 1997, 3: 174–82
D93G	Classic	216	264	14	0.133	0.4	Eur J Hum Genet 1996, 4: 219–24
D93N	Classic	60	131	8	0.073	0.4	J Mol Med 2005, 83: 647–54
D93V	Classic	200	284	12	0.128	0.4	Hum Genomics 2006, 2: 297–309
C94S	Classic	20	19	0	0.031	0.6	Hum Mutat 2001, 18: 459
C94Y	Classic	180	210	0	0.142	0.6	Mol Med 1997, 3: 174–82
A97P	Classic	33	31	0	0.054	9.3	Br J Dermatol 2002, 147: 545–8
A97V	Later-onset	10	12	0	0.025	9.3	Mol Med 1997, 3: 174–82
R100K	Classic	37	22	0	0.043	28.8	Hum Mol Genet 1994, 3: 1795–9
R100T	Classic	205	232	5	0.137	28.8	Mol Med 1997, 3: 174–82
R112C	Classic	26	40	0	0.037	26.1	J Invest Med 2000, 48: 227–35
R112H	Later-onset	70	70	0	0.082	26.1	Hum Mol Genet 1994, 3: 1795–9
R112S	Classic	25	35	0	0.034	26.1	Hum Mutat 2005, 25: 299–305
F113L	Later-onset	3	2	0	0.014	4.4	Mol Med 1997, 3: 174–82
F113S	Hetero	0	0	0	0.005	4.4	Hum Mutat 2001, 18: 459
G138E	Classic	167	203	1	0.121	0.0	Mol Med 2002, 8: 306–12
G138R	Classic	209	259	4	0.277	0.0	Mol Med 1997, 3: 174–82
C142R	Classic	50	89	13	0.065	37.2	Mol Med 1999, 5: 806–11
C142Y	Classic	18	25	9	0.046	37.2	Hum Genet 1995, 95: 557–61
S148N	Classic	21	25	0	0.039	0.0	J Invest Med 2000, 48: 227–35
S148R	Classic	128	177	9	0.104	0.0	Mol Med 1997, 3: 174–82
W162C	Classic	35	35	0	0.045	26.1	Hum Genet 1996, 98: 719–26
W162R	Classic	28	44	0	0.043	26.1	Am J Hum Genet 1993, 53: 1186–97
L166G	Classic	91	101	1	0.081	0.6	Hum Genomics 2006, 2: 297–309
L166V	Classic	14	14	0	0.031	0.6	Hum Genet 1995, 95: 557–61
D170H	Classic	236	302	8	0.120	0.0	Hum Mutat 2003 Sup, 22: 258
D170V	Classic	88	134	4	0.072	0.0	Mol Med 1997, 3: 174–82
G171C	Classic	69	85	7	0.065	4.5	J Dermatol Sci 2008, 52: 61–4
G171D	Classic	88	103	8	0.095	4.5	Hum Mutat 2005, 25: 299–305
G171R	Classic	315	347	12	0.284	4.5	J Mol Med 2005, 83: 647–54
C172F	Classic	12	25	2	0.039	33.5	J Hum Genet 2001, 46: 192–6
C172G	Classic	10	7	2	0.028	33.5	Hum Mutat 2003, 22: 486–92
C172R	Classic	18	45	3	0.049	33.5	J Invest Med 2000, 48: 227–35
C172Y	Classic	12	26	2	0.041	33.5	Hum Mol Genet 1994, 3: 1795–9
G183A	Hetero	35	44	0	0.063	2.1	Biochim Biophys Acta 2010, 1802: 247–52
G183D	Classic	262	296	8	0.204	2.1	Mol Med 1999, 5: 806–811
G183S	Classic	62	103	0	0.088	2.1	Mol Genet Metab 2002, 76: 23–30
M187T	Classic	0	0	0	0.006	0.0	Hum Genomics 2006, 2: 297–309
M187V	Classic	8	13	0	0.030	0.0	J Invest Med 2000, 48: 227–35
S201F	Classic	0	2	0	0.007	6.6	Hum Mutat 2005, 25: 299–305
S201Y	Classic	0	2	0	0.011	6.6	Hum Genomics 2006, 2: 297–309
C202Y	Classic	382	434	12	0.209	0.0	Mol Med 1997, 3: 174–82
C202W	Hetero	276	313	7	0.175	0.0	Hum Mol Genet 1994, 3: 503–5
P205R	Classic	455	571	9	0.257	0.2	Mol Genet Metab 2002, 76: 23–30
P205T	Classic	6	16	0	0.022	0.2	Eur J Hum Genet 1996, 4: 219–24
Y207C	Classic	3	4	3	0.017	50.9	J Inherit Metab Dis 2009, 32: 424–40
Y207S	Classic	4	5	3	0.019	50.9	Mol Genet Metab 2002, 76: 23–30
P210L	Later-onset	0	0	0	0.003	93.3	New case
P210S	Later-onset	0	0	0	0.003	93.3	New case
Y216C	Classic	1	1	0	0.005	7.5	Biochim Biophys Acta 2010, 1802: 247–52
Y216D	Classic	187	246	6	0.145	7.5	Mol Med 1997, 3: 174–82
C223R	Classic	518	589	15	0.255	0.0	Mol Genet Metab 2002, 76: 23–30
C223Y	Classic	451	546	16	0.220	0.0	Mol Genet Metab 2002, 76: 23–30
N224D	Classic	65	67	0	0.057	0.0	Hum Mutat 1998, Suppl 1; S288–90
N224S	Classic	42	47	0	0.054	0.0	J Invest Med 2000, 48: 227–35
D231G	Classic	20	58	3	0.045	54.9	Exp Mol Med 2009, 31: 1–7
D231V	Classic	22	64	3	0.041	54.9	Hum Mutat 2008, 29: 331
D234E	Classic	20	27	1	0.043	40.9	Hum Mutat 2005, 25: 299–305
D234Y	Classic	352	456	13	0.270	40.9	Mol Genet Metab 2002, 76: 23–30
S235C	Classic	0	0	0	0.004	53.9	Mol Med 1999, 5: 806–811
S235F	Classic	0	1	0	0.004	53.9	Hum Mutat 2008, 29: 331
W236C	Classic	2	7	0	0.012	40.6	Eur J Hum Genet 1996, 4: 219–24
W236L	Classic	0	2	0	0.005	40.6	Mol Med 1999, 5: 806–811
W236R	Classic	6	23	0	0.025	40.6	Hum Genomics 2006, 2: 297–309
D244H	Hetero	226	287	5	0.122	74.8	Mol Med 1999, 5: 806–811
D244N	Classic	20	42	0	0.037	74.8	Hum Mol Genet 1994, 3: 1795–9
G258R	Hetero	97	87	0	0.101	0.8	Hum Mutat 2001, 18: 459
G258V	Classic	67	67	0	0.087	0.8	Hum Mutat 2008, 29: 331
D264V	Classic	132	194	9	0.097	11.2	Am J Hum Genet 1993, 53: 1186–97
D264Y	Classic	103	132	6	0.087	11.2	Hum Mutat 2005, 25: 299–305
D266E	Classic	40	69	8	0.061	5.9	Mol Med 2002, 8: 306–12
D266H	Hetero	445	596	16	0.200	5.9	J Investig Med 2000, 48: 227–35
D266N	Classic	48	75	10	0.059	5.9	Clin Genet 2000, 58: 228–33
D266V	Classic	26	43	7	0.039	5.9	Am J Hum Genet 1993, 53: 1186–97
D266Y	Classic	39	79	10	0.047	5.9	Mol Genet Metab 2008, 95: 224–8
M267I	Classic	75	105	16	0.095	4.2	Mol Med 1999, 5: 806–811
M267R	Classic	132	192	15	0.097	4.2	Hum Genomics 2006, 2: 297–309
V269A	Classic	10	18	2	0.029	0.0	Hum Mol Genet 1993, 2: 1051–3
V269M	Classic	113	129	8	0.104	0.0	Hum Genomics 2006, 2: 297–309
G271C	Classic	58	57	0	0.066	0.2	Mol Genet Metab 2002, 76: 23–30
G271S	Classic	45	65	0	0.057	0.2	Hum Genomics 2006, 2: 297–309
G271V	Classic	195	239	7	0.149	0.2	Hum Genomics 2006, 2: 297–309
N272K	Classic	60	100	0	0.065	2.8	Hum Mol Genet 1994, 3: 1795–9
N272S	Classic	2	3	0	0.013	2.8	Eur J Hum Genet 2004, 12: 678–81
Q279E	Later-onset	34	26	0	0.035	20.4	Hum Genet 1992, 89: 29–32
Q279H	Classic	102	123	4	0.094	20.4	Hum Mutat 2001, 18: 459
Q279R	Classic	55	56	0	0.061	20.4	Hum Mutat 2003 Sup, 22: 258
Q280H	Hetero	87	104	0	0.079	0.0	Hum Mutat 2001, 18: 459
Q280K	Classic	30	41	0	0.040	0.0	J Mol Med 2005, 83: 647–54
T282A	Classic	3	0	0	0.008	1.2	Hum Mutat 2008, 29: 331
T282N	Classic	6	9	0	0.023	1.2	J Hum Genet 2001, 46: 192–6
A285D	Classic	32	29	0	0.041	0.0	Hum Genomics 2006, 2: 297–309
A285P	Classic	21	29	0	0.043	0.0	Hum Mutat 2005, 25: 299–305
W287C	Classic	16	14	0	0.028	1.6	Mol Med 1997, 3: 174–82
W287G	Classic	11	12	0	0.026	1.6	Eur J Hum Genet 1996, 4: 219–24
A288D	Classic	86	96	0	0.106	0.0	Hum Mol Genet 1994, 3: 1795–9
A288P	Classic	50	60	0	0.068	0.0	Mol Genet Metab 2002, 76: 23–30
P293A	Classic	75	58	0	0.069	2.6	Mol Genet Metab 2002, 76: 23–30
P293T	Classic	13	18	0	0.032	2.6	Hum Genomics 2006, 2: 297–309
M296I	Later-onset	5	12	0	0.018	0.0	New Eng J Med 1995, 333: 288–93
M296V	Later-onset	7	13	0	0.023	0.0	New Eng J Med 1991, 324: 395–399
S297C	Classic	0	1	0	0.011	0.0	Mol Med 2002, 8: 306–12
S297F	Classic	215	268	8	0.145	0.0	Am J Hum Genet 1993, 53: 1186–97
N298H	Hetero	140	181	0	0.089	0.0	Eur J Hum Genet 1996, 4: 219–24
N298K	Classic	106	183	0	0.092	0.0	Hum Mutat 1996, 8: 38–43
N298S	Classic	18	14	0	0.029	0.0	Mol Med 1997, 3: 174–82
R301Q	Later-onset	112	166	0	0.126	40.5	Am J Hum Genet 1990, 47: 784–9
R301P	Classic	73	94	0	0.074	40.5	J Hum Genet 2001, 46: 192–6
N320K	Classic	150	154	0	0.105	0.0	Biochem Biophys Res Commun 1995, 214: 1219–24
N320Y	Classic	239	286	0	0.183	0.0	J Invest Med 2000, 48: 227–35
Q321E	Classic	124	144	0	0.088	42.6	Mol Med 1999, 5: 806–811
Q321R	Classic	22	24	0	0.034	42.6	Hum Genomics 2006, 2: 297–309
G328A	Classic	145	139	0	0.100	0.0	Am J Hum Genet 1993, 53: 1186–97
G328R	Classic	560	584	4	0.283	0.0	Hum Genet 1992, 89: 29–32
G328V	Classic	222	239	0	0.160	0.0	Hum Mutat 2005, 25: 299–305
E358A	Classic	102	175	0	0.091	90.8	Hum Mutat 2005, 25: 299–305
E358G	Classic	77	136	0	0.083	90.8	Mol Med 2002, 8: 306–12
E358K	Classic	571	661	9	0.293	90.8	Hum Mutat 1998, Suppl 1: S139–40
G373D	Classic	103	116	0	0.116	1.0	Hum Mutat 2001, 17: 353
G373S	Classic	1	0	0	0.004	1.0	Biochem Biophys Res Commun 1995, 214: 1219–24
C382W	Hetero	271	265	0	0.205	0.2	Intern Med J 2002, 32: 575–84
C382Y	Classic	241	245	0	0.203	0.2	Hum Mutat 2003 Sup, 22: 258
P409A	Classic	13	11	0	0.049	37.2	Hum Mutat 2001, 18: 459
P409S	Classic	10	4	0	0.024	37.2	Mol Med 2002, 8: 306–12
P409T	Hetero	21	18	0	0.078	37.2	Hum Mutat 2001, 18: 459
T410A	Later-onset	0	0	0	0.005	18.6	Clin Genet 2003, 63: 205–9
T410P	Classic	77	93	0	0.089	18.6	Hum Mutat 2008, 29: 331

Classic, the classic form of Fabry disease; Later-onset, the later-onset form; Hetero, heterozygote of Fabry disease; and Polymorphism, GLA polymorphism.

### Structural analysis of representative amino acid substitutions

**Figure 1:**
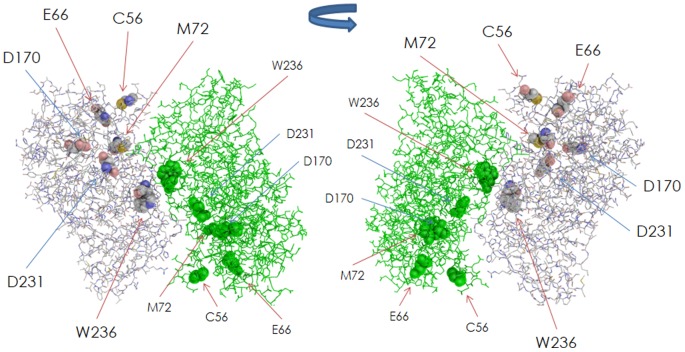
Structure of the GLA dimer and positions of the amino acid residues involved in the substitutions. The backbone is shown as a line. Subunit A and subunit B comprising the dimer are shown in light blue and green, respectively. The amino acids involved in the substitutions (C56, E66, M72 and W236) and the catalytic residues (D170 and D231) are indicated as a CPK model. Front view (left) and back view (right).

We examined different amino acid substitutions at M72, E66, C56, and W236, because they are expected to provide us with useful information for elucidating the mechanism by which structural changes caused by them influence the severity of the disease and for identifying residues essential for the maintenance of proper folding. The localization of these residues in the dimer is shown in Fig. 1. The residues are widely distributed over the GLA molecule and are distant from the catalytic residues (D170 and D231).

#### M72 (M72I, M72R, and M72V)

**Figure 2:**
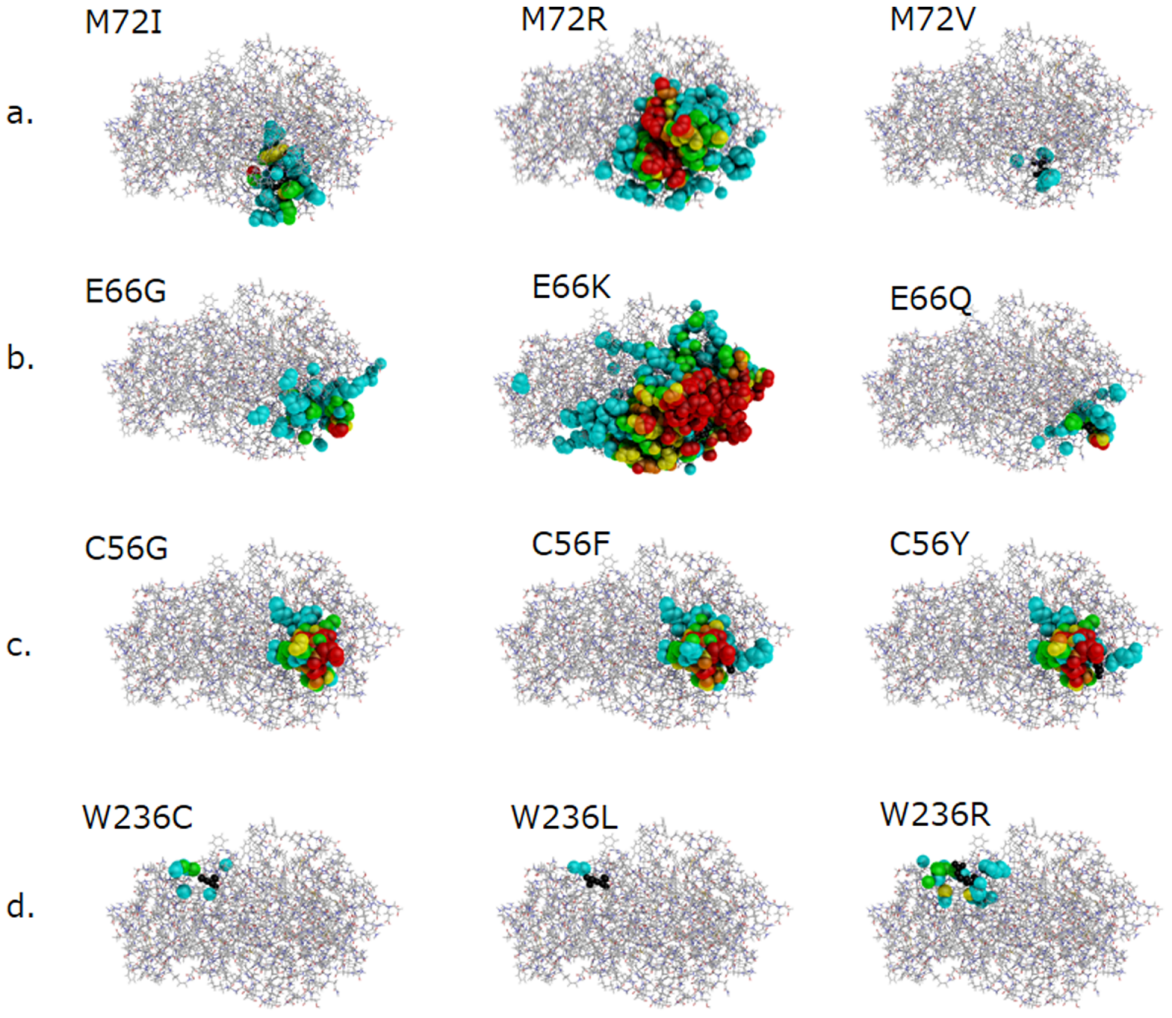
Coloring of the atoms in the three-dimensional structure of GLA influenced by amino acid substitutions at M72 (a), E66 (b), C56 (c), and W236 (d). The backbone of GLA is shown as a line. The atoms of the substituted residues are indicated as small black spheres and the influenced atoms as large spheres. The colors of the influenced atoms show the distances between the wild type and mutant ones as follows: 0.15 Å≤cyan <0.30 Å, 0.30 Å≤green <0.45 Å, 0.45 Å≤yellow <0.60 Å, 0.60 Å≤orange <0.75 Å, and red ≥0.75 Å.

M72 is located on the α-helix (66–84) of the (β/α)_8_ barrel domain. The ASA value of this residue is 0 Å^2^, suggesting that it is fully buried. The numbers of atoms influenced by M72I in the main chain, side chain and active site are 38, 46 and 0, respectively, the RMSD value being 0.054 Å. The numbers of atoms influenced by M72R in the main chain, side chain and active site are 145, 198, and 1, respectively, the RMSD value being 0.119 Å. Considering the results, the structural changes in GLA caused by these amino acid substitutions are thought to be large. The patients with these mutations exhibited the classic form of Fabry disease. On the other hand, as to M72V, the numbers of atoms influenced in the main chain, side chain and active site are 7, 6 and 0, respectively, the RMSD value being 0.026 Å. This suggests that the structural change caused by M72V is small, and that it does not affect the active site. The patients with M72V exhibited the later-onset Fabry disease. Coloring of the influenced atoms allowed clear visualization of the differences in the structural changes between these cases (Fig. 2a).

#### E66 (E66G, E66K and E66Q)

E66 is located on the α-helix (66–84) of the (β/α)_8_ barrel domain. The ASA value is 29.2 Å^2^, suggesting that the residue is half-exposed to the solvent. For the E66G substitution, the numbers of atoms influenced in the main chain, side chain and active site are 45, 74, and 0, respectively, the RMSD value being 0.062 Å. For the E66K substitution, the numbers of atoms affected in the main chain, side chain and active site are 422, 503, and 7, respectively, the RMSD value being 0.361 Å. The patients with such large structural changes exhibited the classic form of Fabry disease. On the other hand, as to the E66Q substitution, which has been reported to be a functional polymorphism [21], the numbers of atoms affected in the main chain, side chain, and active site are 23, 32, and 0, respectively, the RMSD value being 0.048 Å. These results suggest that the structural change is moderate and that it does not affect the active site. Fig. 2b clearly shows that the structural change caused by E66Q is restricted to a small region on the molecular surface, although those caused by E66G and E66K extend over a broad area around the substituted residue.

#### C56 (C56G, C56F, and C56Y)

**Figure 3:**
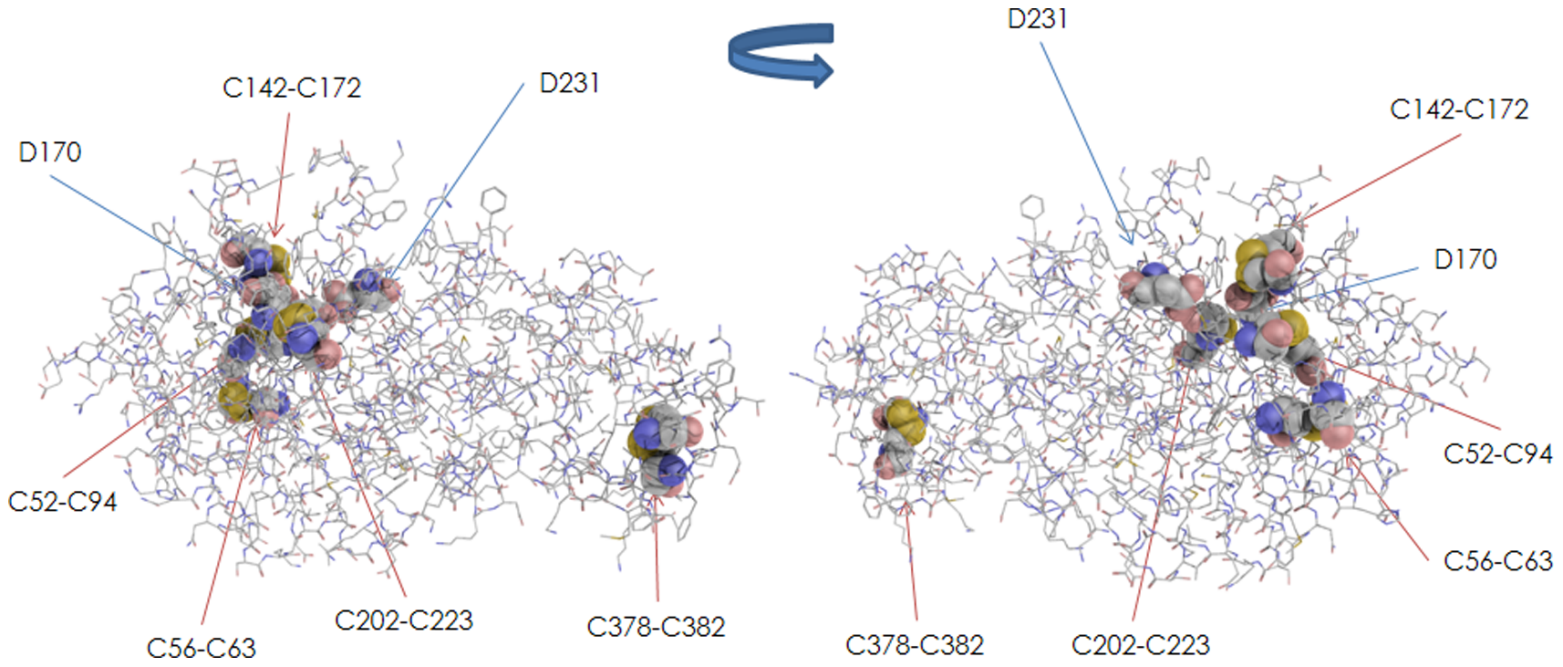
GLA structure and residues involving a disulfide bond. The backbone of GLA is shown as a line. The atoms involved in the formation of a disulfide bond (C52-C94, C56-C63, C142-C172, C202-C223, and C378-C382) and the catalytic residues (D170 and D231) are shown as a CPK model. Front view (left) and back view (right).

C56 is located between two α-helices (47–50 and 66–84). The ASA value of the residue is 38.4 Å^2^, suggesting that it is exposed to the solvent. The C56 residue forms a disulfide bond with C63 (Fig. 3), and it plays an important role in conformation of the enzyme molecule. Fig. 2c shows the structural changes caused by the C56G, C56F, and C56Y amino acid substitutions. These amino acid substitutions at the C56 position are predicted to disturb the formation of disulfide bond between C56 and C63, and thus the mutant proteins would be excessively degraded before they are transported to the lysosomes. All of the patients with these mutations presented the classic form of Fabry disease.

#### W236 (W236C, W236L, and W236R)

**Figure 4:**
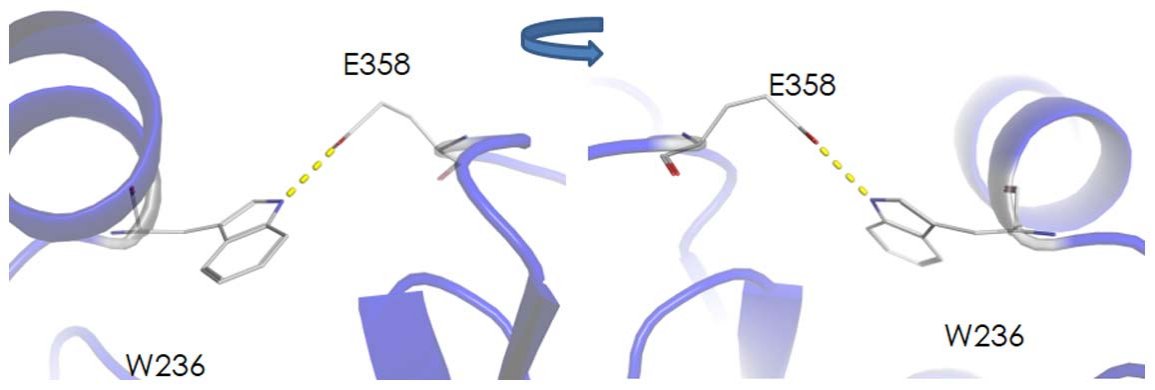
The hydrogen bond between W236 and E358. The side chain of W236 forms a hydrogen bond with E358. The backbone of GLA is shown as a ribbon model, and W236 and E358 are indicated as a stick. The hydrogen bond is shown as a yellow dotted line. Front view (left) and back view (right).

W236 is located on the α-helix (236–247) of the (β/α)_8_ barrel domain, the ASA value being 40.6 Å^2^, suggesting that the residue is exposed to the solvent. As Fig. 2d shows, the structural changes caused by W236C, W236L, and W236R are small (The numbers of atom in the main chain affected by W236C, W236L, and W236R are 2, 0, and 6, respectively, and those in the side chain are 7, 2, and 23, respectively. The RMSD values for them are 0.012 Å, 0.005 Å, and 0.025 Å, respectively). None of them affects the active site. However, as W236 is located on the dimer interface of GLA (Fig. 1), and the side chain of W236 forms a hydrogen bond with E358 (Fig. 4), the amino acid substitution is thought to affect the conformation of the GLA molecule.

## Discussion

Recently, the results of newborn screening revealed a high incidence (1 in ∼1, 250–9,000) of Fabry disease [22]–[24]. As Fabry disease can be treated with recombinant human GLAs [25]–[27], it is very important to understand the basis of the disease and to predict the outcome for patients found on screening. For this purpose, a structural study will provide us with valuable information. Garman and Garboczi reported that there are at least two classes of mutations in GLA that lead to disease progression: those near the active site and those of buried residues distant from the active site that adversely affect the folded state of the molecule, and a mild phenotype tends to be more solvent-accessible than a severe one [6]. Our research group obtained essentially the same results as those of Garman and Garboczi. Our previous study revealed that structural changes in the classic Fabry group were generally large and tended to be localized to the core region or located in the functionally important region including the active site, and that those in the later-onset group were small and localized on the surface of the molecule [8].

As further structural study, we focused on different substitutions at the same residue in the amino acid sequence of GLA, because such specific cases are useful for examining the influence of the severity of the structural changes on the disease progression and for identifying the residues important for the expression of GLA activity.

In this study, we could select 157 amino acid substitutions at 67 residues from two databases, and examined the correlation between the structural changes in GLA and the clinical phenotype. The results revealed that the structural changes leading to the later-onset Fabry disease tend to be smaller than those for the classic Fabry disease, i.e., M72 is buried and E66 is exposed to the solvent, and at both residues, amino acid substitutions causing a small structural change (M72V and E66Q) lead to later-onset Fabry disease or a functional polymorphism, and ones causing a large structural change (M72I, M72R, E66G, and E66K) result in classic Fabry disease. This study also revealed residues important for expression of the GLA activity. A structural change affecting the active site tends to lead to the classic form. C56 and W236 are thought to be involved in the formation of a disulfide bond and the dimer, respectively. Substitutions at these residues should affect proper folding and lead to classic Fabry disease, even if the structural change is small.

In conclusion, we investigated the effects of different substitutions at the same residue in the amino acid sequence of GLA on structural changes in the enzyme molecule and the clinical phenotype. The results revealed that structural changes influence the disease progression. Structural study from such a unique viewpoint is useful for elucidation of the basis of Fabry disease.
